# Editorial: Breeding Crops for Enhanced Food Safety

**DOI:** 10.3389/fmicb.2022.871247

**Published:** 2022-03-18

**Authors:** Maeli Melotto, Wei Zhang, Max Teplitski

**Affiliations:** ^1^Department of Plant Sciences, University of California, Davis, Davis, CA, United States; ^2^Department of Food Science and Nutrition, Illinois Institute of Technology, Chicago, IL, United States; ^3^International Fresh Produce Association, Washington, DC, United States

**Keywords:** breeding crops, food safety, microbial hazard, toxin, human health

The demand for nutritious and safe food will likely increase as the human population is expected to reach 9.1 billion by 2050 along with increasing urbanization. Furthermore, healthy eating, including the consumption of fresh or minimally processed fruits and vegetables, has become increasingly popular as part of an integrated strategy to decrease the risk of serious diseases. Lamentably however, consumption of fresh produce does not reach the minimum daily recommendations. To this end, decision makers around the world now focus on investing into programs that will ensure nutritional security of populations (a term encompassing access to nutrient-dense rather than high-caloric foods). Achieving such nutritional security will require that all nutrient-dense foods are free of contaminants, including human pathogens.

Unfortunately, over the past decades, fresh fruits and vegetables have been associated with a number of high-profile outbreaks of human foodborne illness. Good agricultural practices, good handling practices, good manufacturing practices, and hazard analysis of critical control points remain the cornerstone of food safety management along the production chain (from farm to fork) as part of “multiple hurdle” approaches to limit produce contamination. Fresh or ready-to-eat produce, such as leafy vegetables and fruits, do not undergo thermal processes to inactivate human pathogens. Instead, they are often treated with aqueous solutions containing sanitizing chemicals to reduce potential cross-contamination and promote quality. The lack of an efficient kill step is one of the greatest challenges facing the fresh produce industry. Thus, novel and comprehensive approaches are needed to ensure the safety and quality of freshly consumed produce. One overlooked yet highly promising approach for reducing susceptibility of crops to colonization with human pathogens and toxin-producing organisms is plant breeding.

Thus far, most of the studies on plant interactions with human pathogens have focused on enterobacteria and toxin-producing fungi as they can grow on/in plants given the right conditions. Multiple examples of differential colonization of enterobacteria in several crop species have been reported and comprehensively reviewed by Henriquez et al. Importantly, these studies revealed that commercial cultivars that are more likely to be contaminated with human pathogens (Jacob and Melotto) represent a higher risk for disease outbreaks. When the crop has a narrow genetic basis, incorporating exotic germplasm compatible with commercial cultivars is an excellent alternative to improve crop safety, as in the case of almond (Gradziel).

Internalization and movement of bacterial cells to edible organs is a safety concern. There are several routes for bacterial internalization into plant organs; different bacterial species are likely to be specialized to preferentially use specific routes. Some human pathogens (such as *Salmonella enterica*) can produce mimics of plant hormones, thus creating openings for colonization of plant tissues. Although human pathogen internalization through lateral root junctions and leaf stomata has been confirmed previously, other points of entry are also possible. In cucumbers, for instance, *S. enterica* can more effectively penetrate blossoms and reach the fruit at high percentage than when entering through the roots (Burris et al.).

The foundation for plant breeding is the existence of genetic variability in the system under investigation, resulting in phenotypic variability (Melotto et al.). Variability in the colonization phenotype depends upon genetic factors of both the plant host and the enterobacteria, as well as environmental conditions. On the leaf surface, for instance, venation density (Doan et al.) and stomatal pore size (Jacob and Melotto) are associated with colonization traits of *E. coli* O157:H7 (i.e., leaf surface attachment and internalization). When plant tissues are able to mount strong and robust immune responses, such as rapid generation of reactive oxygen species and nitric oxide, they are less likely to be colonized by human pathogens (Ferelli et al.). However, at least *S. enterica* is able to evade plant immune responses, such as stomatal closure, and invade the plant tissue (Johnson et al.). A mutant screen revealed regions in the *S. enterica* genome that are required to subvert stomatal immunity (Montano et al.); however, their molecular functions are yet to be fully characterized.

An important aspect of plant breeding is the design of reproducible, robust, fast, and relatively easy protocols for screening the desirable phenotype. Ideally, quantification of the phenotype should be validated with multiple methodologies prior to adoption into breeding programs. These considerations are particularly relevant to advance new fields such as the mechanistic understanding of human pathogen colonization of plants. One of the biggest challenges is to quantify bacterial internalization, persistence, and survival rate in leaves, especially when bacterial populations are small. Some efforts toward this direction have been made for *S. enterica* in leaves. Chahar et al. reported that bacterial internalization can be assessed by several methods and should be adapted to specific systems as steps in the procedure (namely surface sterilization) can interfere with the results. Additionally, a modified protocol for bacterial enumeration and recovery for downstream applications is available and can be adapted to different plant tissues (Oblessuc and Melotto).

Edible crops have been bred for millennia, resulting in crops that are essentially unrecognizable compared to their wild progenitors, and are superior in yield, taste, and a number of agronomic traits. Modern plant breeding tools offer an opportunity to explore the feasibility of breeding crops for their reduced susceptibility to human pathogens and toxin-producing organisms. “Breeding for food safety” does not need to be limited by the canons of the gene-for-gene hypothesis. Articles in this eBook identify crop phenotypes that, for a variety of reasons, are less conducive to human pathogens or toxin-producing microbes. They contributed to a significant advancement in the field of breeding crops for enhanced food safety, highlighting the most up-to-date research progresses, and identifying current knowledge gaps and future directions.

## Author Contributions

All authors served as co-editors to the Research Topic and also contributed to, critically read, discussed, and approved this Editorial.

## Conflict of Interest

The authors declare that the research was conducted in the absence of any commercial or financial relationships that could be construed as a potential conflict of interest.

## Publisher's Note

All claims expressed in this article are solely those of the authors and do not necessarily represent those of their affiliated organizations, or those of the publisher, the editors and the reviewers. Any product that may be evaluated in this article, or claim that may be made by its manufacturer, is not guaranteed or endorsed by the publisher.

